# Circadian Variation of Migraine Attack Onset: A Review of Clinical Studies

**DOI:** 10.1155/2019/4616417

**Published:** 2019-08-25

**Authors:** Daniel Baksa, Kinga Gecse, Sahel Kumar, Zsuzsanna Toth, Zsofia Gal, Xenia Gonda, Gabriella Juhasz

**Affiliations:** ^1^SE-NAP2 Genetic Brain Imaging Migraine Research Group, Hungarian Brain Research Program, Semmelweis University, Budapest, Hungary; ^2^Department of Pharmacodynamics, Faculty of Pharmacy, Semmelweis University, Budapest, Hungary; ^3^MTA-SE Neuropsychopharmacology and Neurochemistry Research Group, Hungarian Academy of Sciences, Semmelweis University, Budapest, Hungary; ^4^Department of Physiology and Neurobiology, Institute of Biology, Faculty of Science, Eotvos Lorand University, Budapest, Hungary; ^5^NAP-2-SE New Antidepressant Target Research Group, Hungarian Brain Research Program, Semmelweis University, Budapest, Hungary; ^6^Department of Psychiatry and Psychotherapy, Semmelweis University, Budapest, Hungary; ^7^Neuroscience and Psychiatry Unit, The University of Manchester and Manchester Academic Health Sciences Centre, Manchester, UK

## Abstract

Several studies suggested that migraine attack onset shows a circadian variation; however, there has not been an overview and synthesis of these findings. A PubMed search with keywords “migraine” AND “circadian” resulted in ten studies directly investigating this topic. Results of these studies mostly show that migraine attacks follow a monophasic 24-hour cyclic pattern with an early morning or late night peak while other studies reported an afternoon peak and also a biphasic 24-hour cycle of attacks. The identified studies showed methodological variation including sample size, inclusion of medication use, comorbidities, and night or shift workers which could have contributed to the contradictory results. Several theories emerged explaining the diurnal distribution of migraine attacks suggesting roles for different phenomena including a morning rise in cortisol levels, a possible hypothalamic dysfunction, a circadian variation of migraine triggers, sleep stages, and a potentially different setting of the circadian pacemaker among migraineurs. At the moment, most studies show an early morning or late night peak of migraine attack onset, but a significant amount of studies reveals contradictory results. Further studies should investigate the arising hypotheses to improve our understanding of the complex mechanism behind the circadian variation of migraine attacks that can shed light on new targets for migraine therapy.

## 1. Introduction

Migraine is a serious and debilitating condition with several comorbidities and a global age-standardized prevalence of 14.4% according to a recent systematic analysis [1]. The disease predominantly affects females with a female-male ratio of 3:1 [2]. The most prevalent form is migraine without aura, which is characterized by usually unilateral, moderate, or severe pulsating headache with nausea, vomiting, and photo- and phonophobia. Approximately one-third of migraineurs also show transient neurological deficits associated with their migraine attacks, collectively termed migraine aura (for example, sensory, retinal, or motor symptoms) [2]. While this high prevalence and the high burden associated with migraine motivate research for underlying pathomechanisms, there is still no clear explanation of when and why a migraine attack will occur. Both clinicians and patients have noted the diurnal variation of migraine attack onset and several terms have been used to describe the periodicity of migraine attacks including cyclical migraine, weekend, or nocturnal migraine, suggesting that migraine attacks do not occur randomly but show distinct temporal patterns [3]. A better understanding of the circadian variation of migraine attack onset could provide relevant information for migraine prevention and intervention. For example, a pulsatile press coated drug delivery of sumatriptan succinate for bedtime administration was created to ensure early morning release to prevent migraine attacks after sleep [4].

However, less than a dozen studies examined directly the circadian variation of migraine attack onset, and the synthesis of these findings has not taken place yet. Therefore, in our review we aimed to collect, organize, and synthesize the results of studies regarding the 24-hour variation in migraine attack onset. We also assemble the suggested theories explaining these findings.

## 2. Methods 

A PubMed search was performed with keywords ”migraine” AND ”circadian” in Title/Abstract field to identify studies on the daily distribution of migraine attacks. The search was limited to publications in English and published before the end of August 2018.

After inspecting the retrieved abstracts we selected relevant papers reporting on the 24-hour distribution of migraine attacks and collated research data from those directly investigating the circadian variation of migraine attack onset.

## 3. Results

### 3.1. Literature Search Results

**Figure 1:**
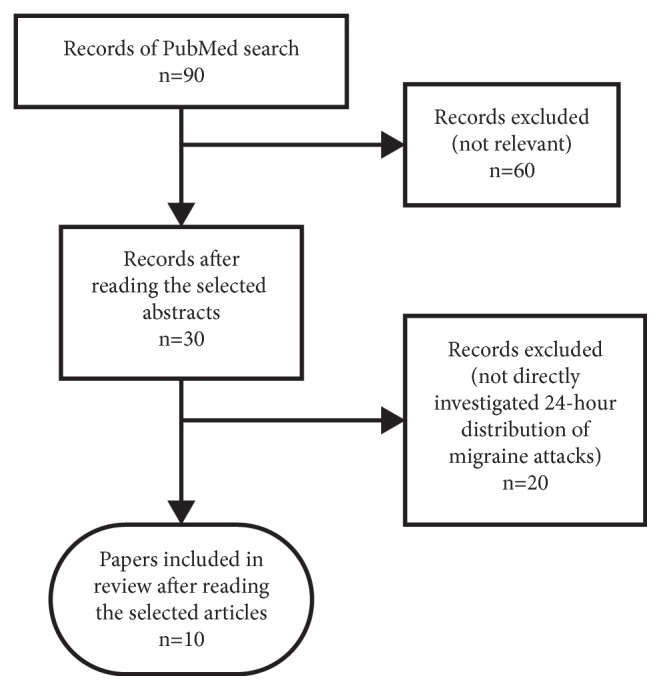
Flowchart of study selection strategy performed after PubMed search for studies directly investigating the circadian variation of migraine attack onset.

Our search identified 90 abstracts. After inspecting the abstracts, we excluded articles discussing other diseases (not migraine) and/or other than circadian aspects of migraine—we selected 30 papers with possibly relevant information on diurnal variation of migraine attack onset. At this stage, we also kept reviews with possible data of original studies. At the final step, after reading the selected papers, 10 studies were identified that directly investigated the circadian variation of migraine attack onset providing new data (for details see Figure 1). In the following we used data from these articles.

### 3.2. Circadian Variation Pattern of Migraine Attack Onset Reported in the Identified Studies

Results of the selected 10 publications are summarized in Table 1. Five studies clearly showed a monophasic 24-hour cyclic pattern of migraine attacks with an early morning or late night peak [5–9], while one study reported one early afternoon peak [3].

The remaining four studies revealed a biphasic 24-hour cycle of attacks. Three of them showed one peak in the morning hours (with a timing similar to that in the majority of the papers that found only one peak), but identified another peaks set at different times: (1) late at night [10], (2) in the afternoon (in a pediatric sample) [11], and (3) just after noon in cases of insomnia-related migraine attacks [12]. The latter study interestingly also showed that attacks unrelated to insomnia peaked only once just after noon [12], which is similar to a finding reported previously by the same research group although in a highly overlapping sample [3]. Another recent article described a peak in the forenoon and another one in the evening, but the authors highlighted that most participants did not report a constant daily distribution of their migraine attacks [13].

### 3.3. Details of the Studies on Circadian Variation of Migraine Attack Onset

Relevant characteristics of the identified studies are shown in Table 1. The majority of studies used a prospective longitudinal design including some with one-year follow-up period, although with much smaller sample sizes compared to those used in one of the two retrospective studies [6] and in a cross-sectional study [10].

The samples mostly consisted of females in their late thirties or forties, although, surprisingly, some studies lack these crucial basic information [7, 9]. One study involved patients from a pediatric population [11]. The effect of gender was adjusted in two studies: Soriani et al. [11] did not find difference between gender groups in a pediatric sample, and van Oosterhout et al. [10] also could not detect any effect of gender. The effect of age was corrected also only in two recent studies: van Oosterhout et al. [10] showed that earlier migraine attack onset (12PM-6AM) was related to higher age, and de Tommaso & Delussi [13] similarly found that migraineurs with prevalent night attack onset (12PM-5AM) were older than the other headache onset groups, except the morning onset group (6AM-12AM). Participants were in most cases migraine patients both with and without aura, but a number of participants belonging to these subgroups are not specified in many articles. Only one study included a sample of chronic migraineurs [13]. For diagnostic criteria every study used a version of the International Headache Society guideline (ICHD) that was available at the time the study was carried out. All the studies used a paper headache diary, except two: Park et al. [8] worked with a smartphone headache diary, and van Oosterhout et al. [10] proceeded with a digital questionnaire.

There is a significant variation between studies regarding medication use in participants. Half of the studies measured sleep quality and/or insomnia [3, 7, 10, 12, 13]. Depression and anxiety symptoms were controlled in only three studies [7, 10, 13]. One study included data on nonmigraine headache besides migraine [8]. Three studies did not provide any information on possible comorbidities [5, 6, 11]. The inclusion of night or shift workers also diverges considerably and three papers [7, 9, 13] even lack this data.

## 4. Discussion

The majority of studies on circadian variation of migraine attack onset showed an early morning or late night peak, while other studies reported an afternoon peak or revealed a biphasic cycle of attacks. Multiple theories have been proposed to explain these results.

### 4.1. Theories Explaining the Circadian Variation of Migraine Attack Onset

The author of the first related study [9] suggested that there is a parallel pattern between migraine, nonfatal myocardial infarction, and sudden cardiac death, all showing a similar circadian rhythm with an early morning peak and sharing other similarities including changes in vasomotor tone and platelet hyperaggregability. Indeed, a higher risk of myocardial infarction has been associated with migraine in a recent meta-analysis [14]. Solomon [9] also noted that platelet hyperaggregability could result in serotonin release potentially contributing to vasospasm which may trigger migraine attacks. Serotonergic neurons can influence the trigeminal system, which has a well-known role in migraine pathophysiology [15], shows increased activity during sleep, and can thus precipitate headaches [16].

Others [6] highlighted that plasma cortisol and adrenocorticotropic hormone (ACTH) levels also show a synchronized zenith in the morning hours. One study [17] reported that migraine patients (compared to headache-free controls) showed higher variation of cortisol level at multiple time periods. Among the investigated migraineurs, subgroups were identified with the following parameters: (1) consistently higher cortisol levels; (2) aberrant pattern of cortisol levels (for example, a rise in cortisol level when all controls and most patients showed a fall); (3) lower cortisol levels at multiple time periods; (4) a similar pattern to controls. Interestingly, the relationship between cortisol level and headache pain was not consistent: only a tendency was found for high cortisol level during pain experience, but severe pain was also reported at normal and low cortisol levels in many cases (and high cortisol level was also addressed during the absence of pain). These results suggest that cortisol, with a peak in the early morning hours, might contribute to some migraine attacks in the morning, but only among some migraineurs. Therefore, it is not surprising that according to a recent review [18] the majority of the studies working with different subgroups of migraineurs and measuring migraine attacks occurring at different times reported that morning cortisol levels did not differ between migraineurs and controls. An interesting prospective longitudinal study applying various stress measures (including morning and evening saliva cortisol) over four days prior to a spontaneous migraine attack onset could not identify a temporal relationship between cortisol changes (or changes in any other stress-related measures) and migraine attack onset [19].

Many other hormones related to migraine also might have a role in circadian variation of migraine attack onset. For example, a study found elevated levels of follicle-stimulating hormone in migraineurs with and without aura (in both sexes) which could be attributed to disturbances in sleep-wake cycle, but no other significant differences were detected in levels of luteinizing hormone, cortisol, and prolactin [20]. Another study with chronic migraine (CM) patients (compared to healthy controls) showed an abnormal pattern of hormones secreted in hypothalamus during the night: a phase-delay in melatonin peak and lower melatonin levels among CM patients with insomnia, suggesting a possible chronobiological dysfunction; a decreased prolactin peak, contributing to a possible hyperdopaminergic state; a higher cortisol concentration; but no difference in growth hormone secretion [21]. Melatonin as a known marker of the circadian clock may be particularly important in understanding the diurnal variation of migraine attack onset. The related studies mostly show lower melatonin levels among migraineurs versus controls [22], but future studies are needed to understand the exact role of melatonin in migraine pathophysiology and its potential effect on attack onset.

Authors of a recent study [8] collected results suggesting that early morning migraine attacks are associated with more severe symptoms (compared to attacks occurring at other times), and perceived pain intensity also shows its maximum early in the morning. They emphasized the role of a possible hypothalamic dysfunction in migraine which may contribute to the circadian variation of migraine attack onset through the hypothalamic involvement in nociception and circadian periodicity. The main circadian pacemaker, the suprachiasmatic nucleus, is located in the hypothalamus [23]. A recent study utilizing high-resolution brainstem imaging with trigeminal nociceptive stimulation showed increased hypothalamic activation in chronic migraineurs compared to headache-free controls, and also among chronic migraineurs compared to episodic migraineurs when participants suffered headache during scanning [24]. This study also revealed higher hypothalamic activation in headache state (among both migraineur types) compared to headache-free state and healthy controls. These results suggest multiple roles for the hypothalamus in migraine: (1) in the initiation of attacks; (2) in acute pain state; and possibly (3) in migraine chronification.

Another theory was proposed by Park et al. [8] who suggested that different trigger factors of migraine occur at different circadian periods, and among them exposure to excessive sleep and sleep deprivation appear most frequently in the morning. However, Alstadhaug et al. [12] reported in an arctic population with significant variations in natural light that insomnia-related attacks peaked in the morning and also just after noon, while attacks not related to insomnia peaked just after noon. Nevertheless, Park et al. [8] notably concluded that circadian variation of migraine attack onset may be a result of interactions between migraine triggers and the innate circadian periodicity. A social nature of migraine attack onset has been also suggested by Alstadhaug et al. [3] suggesting that peak onset in the afternoon might be connected to work-related stress in comparison to morning migraine which might be induced by lack of restorative sleep. Similarly, school activities during the day might contribute to an attack onset peak in the afternoon among children [11].

A temporal relationship between migraine and sleep stages has also been suggested [7]. An EEG study [16] found that migraineurs had minimal sleep disturbance with reduced REM sleep and REM latency. The authors suggested that this connection may be based on shared aminergic mechanisms, for example, through decreased serotonin levels found in both REM sleep [25] and migraine attack [26]. A recent review [27] reported data about the decreased amount of slow wave sleep and a possible involvement of REM sleep in migraine suggesting a probable brainstem dysfunction in networks involved in switching between sleep stages. According to this review studies also show the following sleep characteristics of the night before migraine attacks occur early in the morning or late at night: increased awakenings, decreased amount of slow wave sleep, and insomnia.

A possibly different setting of the endogenous circadian pacemaker in migraineurs has been suggested by van Oosterhout et al. [10]. Self-report questionnaire data showed that migraineurs in comparison to headache-free control subjects were less flexible in adapting to circadian rhythm changes and more prone to show an early or late chronotype, and early morning attacks were related to early chronotype. This hypothesis may be supported by the result showing that migraines with or without aura were connected to a mutation in the casein kinase I*δ* gene (*CKIδ*) (with phosphorylating effect on the Per2 circadian protein) in two families with advanced sleep phase syndrome [28].

### 4.2. Methodological Differences between Studies on Circadian Variation of Migraine Attack Onset

We also need to highlight that the revealed methodological differences may have significantly contributed to the varying results of studies on the diurnal distribution of migraine attacks. As a favourable tendency, most studies used a prospective longitudinal design; however, in most cases this method correlates with a significant reduction of sample size. A recent study [13] represents a good example towards a possibly preferable approach: the researchers employed a prospective design for a shorter period of time (3 months) with a sample consisting of nearly 800 patients (a much higher number compared to other prospective studies). Although, the disadvantage of this strategy is a shorter follow-up time which might bias the results, because migraine attacks may also show seasonal variation. In arctic populations higher frequency of migraine attacks were found during the bright arctic summer (mostly among migraineurs with aura) which is probably connected to the prolonged light exposure that might trigger migraine [29–31]. A study in Italy identified a peak in January and the lowest attack incidence in August [32]. Hospital admissions for migraine have also been detected to show seasonal variation. A study reviewing a 20-year period in a US hospital found that admission of female migraineurs peaks in spring (in comparison to males) [33]. Studies in pediatric headache patients (including migraineurs) showed a higher admission frequency in autumn possibly associated with the beginning of the school year representing a potential stress factor for children with headache [34, 35]. These data indicate the importance of considering the possibility of seasonal variation in migraine attacks in case of a research period spanning less than a year. The climate of the area where the research takes place can also be relevant in this regard: studies performed in different climates and/or with varying investigation periods can produce contrasting results.

Another important aspect is the exact diagnosis of migraine patients. Most of the reviewed studies included samples of migraine patients with and without aura, but their actual numbers (and other details) were not always provided. When such subsample sizes are available, there are large differences in the number of patients in these migraine categories [8, 13]. Therefore results of these studies may not be generalizable among migraineurs with and without aura. Others also highlight that the selected population type (headache center patients versus general population) can also contribute to the divergence between results [13]. Migraineurs represent a heterogenous population, so the use of different subgroups and/or attack types like insomnia-related and -nonrelated attacks such as in the paper of Alstadhaug et al. [12] could also be beneficial: it can help identify specific mechanisms that occur only in a certain migraine subsample or attack type. Although, this approach also weakens the comparability of results between studies employing differently defined migraine subcategories.

Use of allowed medications is a crucial factor in the reviewed studies. For example* beta-blockers *might affect the rhythm of migraine attack onset [9]. The blockage of *β*_1_-receptors is responsible for sleep disturbances possibly caused by reduced nocturnal melatonin release [36]. Concerning* sleep medication*, short-acting benzodiazepines may increase the risk of attacks in a dose-dependent way [37]; meanwhile Z-drug eszopiclone has no influence on migraine [38].* Contraceptives *can also contribute to a different setting of attacks, for example, through changes in hormone level fluctuations. The drop in estrogen level is a trigger for women suffering from menstrual migraine. In that case, hormonal treatment could be preventive [39]. However, oral contraceptives can modulate some circadian rhythm parameters (e.g., blood pressure, heart rate, skin blood flow), although without a significant effect on the circadian clock itself [40]. Nonsteroidal anti-inflammatory drugs (*NSAIDs)* like naproxen show no significant changes in circadian patterns of ACTH and cortisol in healthy volunteers [41]. However, it was shown [42] that the effect of other NSAIDs on ACTH and cortisol levels depends on treatment duration. After a singular intake of indomethacin or acetylsalicylic acid (ASA), no changes were detected, while after a 4-day treatment, both medications resulted in changes in stress hormone level. In insulin induced hypoglycemia stress test, indomethacin intake resulted in increased ACTH and decreased cortisol levels, while in case of ASA administration, ACTH levels decreased and cortisol levels remained unaltered. These examples illustrate the need to control for medication use in studies of circadian variation of migraine attack onset.

Migraine has several comorbidities including ones connected to circadian dysregulation, such as asthma, insomnia [23], major depressive disorder, and bipolar disorder [43]. Comorbid illnesses can influence migraine symptoms. For example, migraineurs with comorbid psychiatric diseases have higher attack frequency compared to migraineurs without psychiatric comorbidities [44]. These data highlight the need to control for comorbidities in studies on circadian variation of migraine attack onset. However, only a small amount of the reviewed studies employed data on comorbidities, mostly on sleep-related ones and anxiety and depression symptoms in a few cases. Environmental factors also have a known role in migraine, including stress [45, 46] which has also been associated with circadian rhythms through an interaction between the hypothalamic-pituitary-adrenal axis and the autonomic nervous system, both involved in stress regulation and provided with circadian inputs [47]. Therefore, it would be beneficial to control for environmental factors in studies of circadian variation of migraine attacks.

Night or shift work can result in a circadian disruption with a potential carcinogenic effect [48]. Sleep disturbances are known triggers of migraine [49], and a study from Denmark showed a higher prevalence of treatment-seeking migraine patients among evening workers [50]. These data suggest that shift workers should be investigated separately from (or in comparison with) nonshift workers in studies of circadian variation of migraine attack onset.

## 5. Limitations

Only published articles (until August 2018) from English-language journals were searched; data from conference abstracts or publications written in other languages were not included. We did not have information on unpublished data or on any form of publication bias.

We identified other studies that investigated a similar subject, but did not employ a full 24-hour long time range regarding the distribution of migraine attacks; therefore such articles were left out from the current review.

## 6. Conclusion

Most studies on circadian variation of migraine attack onset show an early morning or late night peak but there is a significant amount of studies revealing contrasting data. This contradiction could at least in part result from methodological differences between studies. Heterogeneity among migraineurs and migraine attacks is another option possibly contributing to discrepancy, as different migraine subtypes may develop via different pathways in their pathomechanism, and migraine attacks can be provoked by several factors. Future studies should focus on producing more generalizable data with the use of large enough samples (even among migraine subtypes) and controlling for all the relevant determinants, including age, gender, comorbidities, and environmental factors, especially stress. For the present, we only have theories that need to be tested in future studies as the exact mechanism explaining the circadian variation of migraine attack onset is not fully understood yet. A better understanding of this complex mechanism could contribute to the development of migraine therapy.

## Figures and Tables

**Table 1:** Details and results of the studies on circadian variation of migraine attack onset.

Study	Design	Time-span	Sample	Age (years) [*adjustment for effect*: *∗*]	Female [*adjustment for effect*: *∗*]	Migraine type (sample size if available)	Medication	Comorbid factors	Night work/shift work	Circadian variation of attack onset (peak)
*Studies showing a monophasic 24-hour cyclic manner of migraine attacks*										

Solomon (1992) [9]	prospective	20 weeks	15 patients (USA)	no data	no data	MO, MA	NSAID or placebo used (beta-blockers excluded)	healthy (except migraine)	no data	6_AM_-12_AM_

Fox & Davis (1998) [6]	retrospective	last 3 years	1698 patients (USA)	from 18 to 60+	89%	MO, MA	use of oral contraceptives included	no data	included	4_AM_-9_AM_

Fox (2005) [5]	literature data analysis	-	1698 patients (USA)	from 18 (median: 34)	~4:1	MO, MA	excluded w. oral contraceptives	no data	not included	1_AM_-6_AM_

Gori, et al. (2005) [7]	retrospective	last 3 months	100 patients (Italy)	patients (23-50), mean: 38.6; control (23-60), mean: 37.1	no data	MO	no data	depression and anxiety symptoms; sleep quality	no data	3_AM_-7_AM_

Alstadhaug, et al. (2008) [3]	prospective	12 months	84 patients (arctic)	mean ~35	100%	MO (34), MO+MA (50)	use of oral contraceptives, beta-blockers included	healthy (except migraine); insomnia	included	at 1.40_PM_

Park, et al. (2018) [8]	prospective	90 days	82 patients (Korea)	mean 37.4	84.1%	MO (81), MA (1)	use of acute & preventive medication allowed	non-migraine type headache	not included	6_AM_-12_AM_

*Studies showing a biphasic 24-hour cyclic manner of migraine attacks*										

Soriani, et al. (2006) [11]	prospective	12 months	115 patients (pediatric) (Italy)	5-18 yrs (median: ~10)	47% [*∗*]	MO	excluded if taking prophylactic medication	no data	-	main peak at 4:48_PM_, secondary peak at 6:35_AM_

Alstadhaug, et al. (2007) [12]	prospective	12 months	68 patients (arctic)	mean 35.5	100%	MO, MA (insomnia-related (29%) & not related attacks)	excluded if taken beta-blockers	possible chronic insomnia; sleep quality on the night prior to or the night of the attack	not included	insomnia related: peak early in the morning (~8_AM_) & just after noon/not related: peak just after noon

van Oosterhout, et al. (2018) [10]	cross-sectional	-	2389 patients, 189 controls (Dutch)	mean 45.2 [*∗*]	83.4% [*∗*]	MO, MA	use of sleep medication (no data on migraine medication)	depression and anxiety symptoms, lifetime depression; sleep quality	included	peaks: from midnight to 6_AM_ (34.5%) & from 6_AM_ to noon (31.7%)

de Tommaso & Delussi (2018) [13]	prospective	3 months	786 cases (Italy)	means: MO – 37.4; MO+MA – 34.2; CM – 42.5 [*∗*]	~80%	MO (538), MO+MA (52), CM (196)	exclusion: use of central nervous system-active drugs or preventive treatment for primary headache	healthy (except migraine); depression and anxiety symptoms; sleep features; quality of life	no data	two peaks: at 10_AM_ & at 10_PM_ (but most patients did not report a constant circadian rhythm of their attacks)

Table 1 shows the results and other important details of the selected studies on circadian variation of migraine attack onset. Abbreviations. CM: chronic migraine; MA: migraine with aura; MO: migraine without aura; NSAID: nonsteroidal anti-inflammatory drug; *∗*: adjustment for effect.

## Abbreviations

ACTH: Adrenocorticotropic hormone
ASA: Acetylsalicylic acid
*CKIδ*: Casein kinase I*δ* gene
CM: Chronic migraine
MA: Migraine with aura
MO: Migraine without aura
NSAID: Nonsteroidal anti-inflammatory drug.
